# Perinatal maternal life events and psychotic experiences in children at twelve years in a birth cohort study^☆^

**DOI:** 10.1016/j.schres.2013.11.006

**Published:** 2014-01

**Authors:** Sarah Dorrington, Stan Zammit, Laura Asher, Jonathan Evans, Jonathan Heron, Glyn Lewis

**Affiliations:** a Department of Psychological Medicine, Institute of Psychiatry, King's College London, London SE5 8AF, United Kingdom; b School of Social and Community Medicine, Oakfield House, Oakfield Grove, Bristol BS8 2BN, United Kingdom; c Centre for Global Mental Health, London School of Hygiene and Tropical Medicine, Keppel Street, London WC1E 7HT, United Kingdom; d Department of Psychological Medicine & Neurology, MRC Centre for Neuropsychiatric Genetics and Genomics, Cardiff University, Heath Park, Cardiff CF14 4XN, United Kingdom; e Mental Health Sciences Unit, Faculty of Brain Sciences, University College London, W1W 7EJ, United Kingdom

**Keywords:** HPA, hypothalamic–pituitary–adrenal axis, Psychosis, Schizophrenia, Life events, HPA axis, Perinatal psychiatry, Child psychiatry, ALSPAC

## Abstract

**Background:**

International studies indicate that the median prevalence of psychotic experiences in children is 7%. It has been proposed that environmental stress during pregnancy may affect the neurodevelopment of the foetus and lead to a vulnerability in the child to later stressors and psychopathology.

**Aim:**

In this study we explore the relationship between environmental stress during pregnancy and psychotic experiences in children in the general population at 12 years.

**Methods:**

We analysed a birth cohort of 5038 children from the Avon Longitudinal Study of Parents and Children. Environmental stress was measured as life event exposure. Data on life events were collected on women during their pregnancy, whilst psychotic experiences in the offspring were assessed at age 12.

**Results:**

There was a weak association between maternal exposure to life events and psychotic experiences at twelve years (crude OR 1.10 95% CI 1.02–1.18) per quartile of life event score. This association was not reduced after adjustment for socio-economic status, family history of schizophrenia, maternal education or birth weight but after adjustment for maternal anxiety and depression and smoking in early pregnancy there was no longer any evidence for an association (OR 1.01 95% CI 0.93–1.10).

**Conclusion:**

This study provides some evidence to suggest that stressful life events may affect child psychotic experiences through effects on maternal psychopathology, and possibly physiology, during pregnancy.

## 1 Introduction

It has been proposed that environmental stress, during pregnancy, such as life event exposure, may affect the neurodevelopment of the foetus and lead to an increased risk of psychopathology (Lou et al., 1994, Walker and Diforio, 1997, van Os and Selten, 1998, Araya et al., 2009, Kinsella and Monk, 2009).

Studies looking at the effects of stress on foetal development suggest that glucocorticoids play a role in both early neurodevelopment and later atrophic processes which may be involved in the development of a range of psychopathology (Cotter and Pariante, 2002), including psychotic experiences in childhood. Neurodevelopmental studies have found that prenatal environment can alter behaviour and neuroendocrine systems, and affect the hippocampal structure of primates (Coe et al., 2003). Reduced hippocampal formation (Weinberger, 1999) and variation in pituitary volume (Garner et al., 2009) have also been seen throughout the progression of schizophrenia in humans.

Several studies propose that these alterations in the hypothalamic–pituitary–adrenal (HPA) axis (Gitau et al., 1998, Kaplan et al., 2008, O'Donnell et al., 2009) and alterations in placental enzymes (Gluckman et al., 1999) caused by environmental stress may affect the neurodevelopment of the foetus (Coe et al., 2003) and have significant later effects on the child (Talge et al., 2007) Walker and Diforio's (1997) neural diathesis stress model of schizophrenia incorporates prenatal factors and the augmentation of dopamine and dopamine receptor synthesis by the HPA axis (Walker and Diforio, 1997). Longitudinal studies are required to examine effects of prenatal stressors on risk of schizophrenia whilst minimizing recall bias, but are rarely feasible given the incidence of this disorder. However, psychotic experiences during childhood are relatively common in the general population and offer the potential for using cohort studies to examine the aetiology of psychosis. In the Avon Longitudinal Study of Parents and Children (ALSPAC) cohort, definite psychotic experiences were present in approximately 5% of children aged 12 years (Horwood et al., 2008). This finding is consistent with findings from a recent systematic review and meta-analysis that reported the median prevalence of psychotic experiences as 7% (Linscott and van Os, 2012). Although it is not clear to what extent psychotic experiences reflect pathology underlying disorders such as schizophrenia, and the majority of such experiences in childhood remit (Escher et al., 2002, van Os et al., 2009, De Leede-Smith and Barkus, 2013), nevertheless children with psychotic experiences are at increased risk of developing schizophreniform disorder (Poulton et al., 2000) and other psychotic disorders (van Os et al., 2009, Werbeloff et al., 2012, Zammit et al., 2013) in later life.

Although Spauwen et al. reported an association between psychotic experiences in children and antenatal life events (Spauwen et al., 2004), this relationship has not been adequately explored. In their study of 963 adolescents, children exposed to antenatal or pregnancy-related stress had increased odds of reporting psychotic experiences in adolescence, but the main limitation of this study was that data was retrospective, and therefore results may have been affected by maternal recall bias.

The aim of this study is to determine, using longitudinal data, if there is an association between perinatal environmental stressors, measured as life event exposure during pregnancy, and psychotic experiences at twelve years in a birth cohort study.

## 2 Methodology

### 2.1 Sample

Data were drawn from the Avon Longitudinal Study of Parents and Children (ALSPAC), an ongoing population-based study designed to investigate the effects of a wide range of influences on the health and development of children. Pregnant women residing in the south-west of England who had an estimated date of delivery between April 1, 1991 and December 31, 1992 were invited to participate. The initial study cohort consisted of 14,062 pregnancies and 13,978 (52% boys and 48% girls) singletons/twins still alive at 12 months of age.

Parents have completed regular postal questionnaires concerning their child's health and development since birth. Since 7.5 years of age, the children attended annual assessment clinics where they participated in face-to-face interviews. In this study, lower social class families, parents with lower education, male children and minority ethnic status participants were more likely to be non-attenders (Horwood et al., 2008).

Compared to the 1991 UK National Census Data, the ALSPAC sample showed a slightly higher proportion of house owner–occupiers and a smaller proportion of mothers from ethnic minorities (Golding et al., 2001). As described in Boyd et al. (2013), children enrolled in ALSPAC were more educated at 16 compared to the national average, were more likely to be white (reflecting the ethnical composition of the area from which the sample was drawn) and less likely to be eligible for free school meals (an indicator of low income in the UK). Detailed information about ALSPAC is available online (http://www.bris.ac.uk/alspac). Please note that the study website contains details of all the data that is available through a fully searchable data dictionary.

The ‘psychotic symptoms in childhood semi-structured interview’ (Horwood et al., 2008), was conducted when children were 12 years of age.

### 2.2 Measures

#### 2.2.1 Outcomes

The interview for psychotic experiences in childhood consists of 12 core questions covering the last 6 months. Questions include visual and auditory hallucinations; delusions of being spied on, persecution, thoughts being read, reference, control, grandiose ability and other unspecified delusions; and experiences of thought interference such as thought broadcasting, insertion and withdrawal. For these 12 core items, 7 screening questions were derived from DISC–IV (Shaffer et al., 2000) and 5 questions from the Schedules for Clinical Assessment in Neuropsychiatry (SCAN) version 2.0, modified slightly after piloting. Clinical cross-questioning and probing were used to establish the presence of psychotic experiences, and coding of all items followed the glossary definitions and rating rules for SCAN. Interviewers were psychologists trained in using the psychotic experiences in childhood interview. Psychotic experiences were rated as either not present, suspected or definitely present. Psychotic experiences were only rated as definite when a credible example was provided and unclear responses were always ‘rated down’. Psychotic experiences were included in our analyses if they were not attributable to effects of sleep, fever or substance use. This is consistent with the approach of classification systems for diagnosis of functional psychotic disorders. The average kappa value for interrater reliability was 0.72 (Horwood et al., 2008).

We examined two childhood outcomes:

1) Presence or absence of any suspected or definite psychotic experiences (prevalence 13.2% in total sample at 12 years)
2) A narrower outcome of definite psychotic experiences only (prevalence 5.6% in total sample at 12 years)

#### 2.2.2 Exposures

Data on life events (SLEs) were obtained from a maternal questionnaire delivered at two time points. The sources for the life event questionnaire were the work of Brown et al. (1973a) and Barnett et al. (1983). The questionnaire includes a life-event inventory, which was derived for the present study using previous inventories as a basis for selection of items. Each life event had 5 response categories indicating not only whether or not the event occurred but also to what extent the respondent was affected by it. The early pregnancy questionnaire was given to pregnant mothers at 18 weeks following last menstrual period (LMP). This specifically asked about events in early pregnancy. The late pregnancy questionnaire was given to the same mothers at 8 weeks into the postnatal period. This asked specifically about life events in late pregnancy and postnatally. In addition to our primary analysis we will look at life event data from the early and late pregnancy questionnaires separately. Of completed questionnaires, 40% of early and late pregnancy questionnaires were completed at the specified time points (18 weeks gestation and 8 weeks postnatal). 90% of early pregnancy questionnaires were completed by 25 weeks gestation. 90% of late pregnancy questionnaires were completed by 12 weeks since delivery. 97.1% of mothers who completed the early pregnancy questionnaire completed it by 40 weeks after their LMP (by which time 73.8% of mothers had given birth). This questionnaire is much more specific to pregnancy than the late pregnancy questionnaire. The late pregnancy questionnaire predominantly covers the second half of pregnancy as well as the immediate postnatal period.

If a life event had not occurred then it was scored as 0. For each life event recorded as having occurred, mothers gave the event a subjective scoring from ‘not affected at all’ (1) to ‘severely affected’ (4). In order to take into account the wide variation of severity of life events, and the limited (1–4) subjective ratings, we weighted the life events which affected mothers most severely.

### 2.3 Confounders

Various sociodemographic variables were considered as potential confounders: parental social class (highest of both parents, based on occupation using the 1991 Office for Population Censuses and Surveys classification); gender; and maternal education (four levels ranging from the lowest UK school-leaving qualifications to degree level). We also examined single parenthood; father's age at conception; family history of schizophrenia (in parents and grandparents), family history of depression (in parents and grandparents), maternal history of severe depression, pregnancy complications (gestational diabetes or maternal hypertension) and birth weight as potential confounders. We looked at maternal depression during pregnancy (using the Edinburgh Postnatal Depression Scale) and anxiety during pregnancy (using the Crown–Crisp score).

## 3 Ethical approval

Ethical approval for the study was obtained from the ALSPAC Ethics and Law Committee and the Local Research Ethics Committees.

## 4 Statistical analysis

### 4.1 Weighting the life event exposure

The method for weighting life events was chosen a priori. As shown in Table 1 we divided 40 life events into three groups according to the percentage of mothers who had reported each event as affecting them severely. This was done to account for the wide variation in life events in the life event inventory. The overall severity of each life event was then multiplied by mothers' subjective rating. Subjective scores were multiplied by 1 if < 20% of mothers reported being severely affected, multiplied by 2 if 20–30% of mothers were severely affected, or 3 if > 40% of mothers were severely affected. Combined scores were then made for each mother across both questionnaires, using the weighted subjective scores of each reported event.

**Table 1:** Life events according to reported severity of life event.

40% or more severely affected	No. with each event	20%–39% severely affected	No. with each event	Less than 20% severely affected	No. with each event
Life partner died	32	Friend/relative died	3153	Child ill	3798
Child died	24	Admitted to hospital	6213	Partner ill	2733
Partner rejected child	639	Divorced	205	Friend/relative ill	3985
Separated	889	Very ill	1716	Partner had problems at work	4767
Homeless	313	Mother lost job	803	Problems at work	2434
Attempted suicide	33	Convicted of an offence	52	Argument with partner	12,169
Miscarriage scare	2601	Income reduced	7786	Argument with friend/family	3870
Problem with test on child	751	Moved house	2640	Started a new job	727
Found out having twins	679	Physically hurt by partner	416	Had a test for abnormality	8101
Attempted abortion	145	Major financial problem	3105	Took an exam	1206
Partner emotionally cruel to mum	1454	Event occurred that might harm child	1588	Burgled	1475
		Partner emotionally cruel to child	190	Had an accident	852
		Partner physically hurt child	34		
		Partner lost job	1660		
		Partner in trouble with law	525		
		Partner went away	1968		
		Partner in trouble with the law	361		
					
Weighting = Subjective score × 3		Weighting = Subjective score × 2		Weighting = Subjective score × 1	

We collapsed the combined weighted scores into quartiles. Combined weighted life event scores in the 1st quartile ranged from 0 to 11, in the 2nd quartile from 12 to 20, in the 3rd quartile from 21 to 113, and in the 4th quartile from 114 to 197. Hence, the 1st quartile represents exposure to a lower number of life events and life events of lower severity, and the 4th quartile represents exposure to a greater number of life events and life events of greater severity.

As a check to ensure that results were not unduly influenced by our method of weighting. We compared results of SLEs using our weighted life event score (weighted score) with:

A combined score made from mother's unweighted subjective scores (unweighted score)
A combined score that did not include the subjective score, and had no weighting (binary score). The binary score was made using 0 = no life event and 1 = life event for each of the 44 life events.

### 4.2 Logistic regression

Logistic regression was used to calculate odds ratios (ORs) and 95% confidence intervals (CIs) for psychotic experiences at 12 years given the life event exposures, before and after adjustment for potential confounders. Analyses were conducted using STATA (version 11).

## 5 Results

### 5.1 Final sample

9363 mothers completed both life event questionnaires. 4673 mothers and their children had both exposure and outcome and were included in the final analysis. There were 666 children (13.2% of our sample) with suspected or definite psychotic experiences at 12 years. There were 280 children (5.6%) with definite psychotic experiences.

Table 2a shows the distribution of group characteristics, comparing those with combined weighted life event score in quartiles one, two or three with those with the top quartile. Women with more life events (quartile 4) were more likely to smoke and drink alcohol in pregnancy and have a history of depression compared to women with fewer life events (quartiles 1 to 3). Women with more life events were also more likely to have a male child or a child with low birth weight. Women with more life events were also more likely to report anxiety or depression during pregnancy compared to women with fewer life events.

**Table 2:** Distribution of characteristics in analysis sample.

Group	Potential confounder (n)	% with life event scores in quartiles 1–3 (n) 76.2 (7134)	% with life event scores in quartile 4(n) 23.8 (2229)	p value (chi squared test)
Gender	Male	51.1 (3647)	53.7 (1197)	0.03
Socioeconomic status	Black and minority ethnic	1.7 (114)	2.9 (62)	< 0.001
	Lowest income	42.8 (2897)	43.6 (889)	0.5
	Maternal highest educational achievement 0 level or lower	63.0 (4375)	61.2 (1310)	0.1
Substance use	Smoking in pregnancy	18.9 (1347)	31.7 (707)	< 0.001
	Daily alcohol in pregnancy	1.2 (84)	3.1 (68)	< 0.001
Family history mental health problems	Family history depression	22.1 (1367)	36.0 (642)	< 0.001
	Family history schizophrenia	0.5 (35)	0.8 (17)	0.11
Antenatal complication	Gestational diabetes	0.9 (37)	1.2 (18)	0.3
	Hypertension during pregnancy	7.7 (316)	9.6 (146)	0.02
Birth	Low birth weight (< 2500 g)	3.8 (271)	6.5 (145)	0.02
Maternal antenatal mental health	Maternal anxiety (CC)	10.2 (717)	29.7 (650)	< 0.001
	Maternal depression (EPDS) > 12 18 weeks	7.4 (519)	26.8 (588)	< 0.001
Parental age	Father's age	6.70 (333)	128 (9.10)	0.002
	Mother's age	1.05 (75)	1.62 (36)	0.03

### 5.2 Univariable analysis

In the univariable analysis the association between life events and psychotic experiences was not substantially attenuated when individually adjusted for social class, maternal age, paternal age, gestational diabetes, hypertension during pregnancy, or family history of schizophrenia, and these were therefore not included in the multivariable model, to limit the amount of missing data.

### 5.3 Multivariable analysis

In the multivariable analysis, there was minimal change in the association between life event exposure and psychotic experiences (OR for trend = 1.10 95% CI 1.02–1.18) when we adjusted for sex, ethnicity, maternal education, and birth weight (Table 3, Model A). However, the association between LE and psychotic experiences was eliminated when additionally adjusting for maternal anxiety (Crown–Crisp score) in early pregnancy (OR 1.01 95% CI 0.93–1.09) or depression (EPDS score) in early pregnancy (OR 1.02 95% CI 0.94–1.10). Similarly, adjusting for maternal history of severe depression prior to pregnancy and smoking in the first 3 months of pregnancy also attenuated the association between LE and psychotic experiences (Model B; fully adjusted OR for trend = 1.01 95% CI 0.93–1.10).

**Table 3:** Life event quartiles and psychotic experiences age 12 years, adjusted models A and B.

Life event score quartile	N	% psychotic symptoms^a^ at 12 years	Crude OR (95% CI) p N = 5038	Crude OR (95% CI) Model A N = 4892	Adjusted OR (95% CI) Model B N = 4673
1	1357	12.4	1.0^b^	1.0^b^	1.0^b^
2	1228	12.0	0.96 (0.76–1.22)	1.00 (0.79–1.27)	0.97 (0.76–1.24)
3	1334	13.0	1.06 (0.85–1.33)	1.09 (0.87–1.37)	0.97 (0.76–1.23)
4	1119	15.8	1.32 (1.06–1.67)	1.34 (1.06–1.69)	1.04 (0.81–1.35)
Linear term	5038	13.2	1.10 (1.02–1.18) p = 0.01	1.10 (1.02–1.18) p = 0.01	1.01 (0.93–1.10) p = 0.8

Model A: adjusted for birth weight, maternal education, ethnicity and sex.

Model B: adjusted for previous maternal anxiety 18 weeks gestation, maternal depression 18 weeks gestation, maternal history of severe depression prior to pregnancy and smoking during pregnancy.

a Suspected or definite.

b Reference category.

### 5.4 Comparing early and late life event questionnaires

SLEs rated during the early pregnancy questionnaire were more strongly associated with psychotic experiences at 12 years (OR 1.15, 95% CI 1.07–1.23), compared to those rated during the late pregnancy questionnaire (OR 1.06, 95% CI 0.99–1.13), although confidence intervals overlapped. For both early and late pregnancy questionnaire data there was no evidence of an association between maternal life events and psychotic experiences at 12 years in the fully-adjusted model (B).

### 5.5 Comparing weighted and unweighted life event scores

There was no substantial change in the strength of the association between life event exposure and psychotic experiences when either unweighted scores or binary scores were used in place of weighted scores, or when using weighted scores which were not collapsed into quartiles. Irrespective of score weighting, no association was found between life event exposure and psychotic experiences once early pregnancy anxiety and depression were adjusted for.

### 5.6 Comparing suspected or definite psychotic experiences

There was a slightly larger effect size when examining only definite psychotic experiences as the outcome (OR 1.14 95% CI 1.03–1.28 p = 0.01), in contrast to suspected or definite psychotic experiences (OR 1.10 95% CI 1.02–1.18, p = 0.01). However the estimate was less precise as the numbers with this outcome were smaller.

## 6 Discussion

Are life events during pregnancy associated with psychotic symptoms in offspring at 12 years?

In our unadjusted model, women who experienced a greater number of stressful life events during pregnancy were more likely to have a child who reported psychotic experiences at age 12 years than women with fewer life events. This was not explained by markers of socio-economic position, or family history of schizophrenia. However this association was eliminated after adjusting for maternal anxiety and depression. One interpretation of this finding is that the effects of SLEs are mediated through maternal psychopathology.

The effects of anxiety and depression on psychotic symptoms may occur through both antenatal and postnatal pathways. Prenatal effects of depression on offspring have been found. For example, in a recent study babies born to mothers with depression during pregnancy were found to have increased adrenocorticotrophic hormone levels at birth (Marcus et al., 2011). Research suggests that antenatal depression also disrupts the development of pathways that sensitise the mother to the child's needs postnatally (Pearson et al., 2009, Pearson et al., 2010). Maternal anxiety and depression both during and after pregnancy may disrupt the mother–child relationship, affecting development in the postnatal period and predisposing to psychotic experiences in childhood.

Whilst we believe that the most likely explanation of our findings is that the effects of SLEs are mediated through maternal psychopathology, it is also possible that maternal anxiety and depression are confounding the relationship between SLEs and psychosis. Maternal depression may lead to an increase in reporting or exposure to SLEs, though evidence for this is weaker than for a causal effect of SLEs on depression (Brown et al., 1973b, Kendler et al., 1999). Previous studies have linked socio economic status, personality traits, and genetic pre-determinants to life events (Thapar et al., 1998, Rice et al., 2009). In this study the association between maternal life event exposure and childhood psychotic experiences persisted after adjustment for markers of socioeconomic status.

Smoking during pregnancy also attenuated the association between life events during pregnancy and psychotic symptoms at 12 years. Whilst the characteristics of women who continue to smoke during pregnancy are likely to differ substantially from non-smokers and may be associated both with exposure to life events and risk of offspring psychosis, there is also some evidence to support a causal effect of maternal smoking on offspring psychotic experiences (Zammit et al., 2009a, Zammit et al., 2009b). However, similarly to anxiety and depression during pregnancy, it is not possible to determine from our data whether smoking is a confounder or a mediator of the relationship between life events and psychotic experiences.

Adjusting for other potential confounders made little difference to the results, consistent with findings from other studies. For example Spauwen et al.'s retrospective study found an association between antenatal adverse events and adolescent psychotic experiences that persisted after adjusting for child's gender, socio-economic status (a combination of social status and financial status) and any DSM-IV psychiatric diagnosis in the mother (Spauwen et al., 2004). Despite our adjustment for confounders, the possibility of residual confounding remains.

Large cohort studies have found an association between stressful life events in pregnancy and later schizophrenia (Khashan et al., 2008, Malaspina et al., 2008). However, the effect of maternal mental health as a potential confounder or mediator of this association was not examined in these studies.

Psychotic experiences in general population samples frequently co-occur with depression and it is not clear to what extent psychotic experiences in the general population reflect pathology underlying psychotic disorders such as schizophrenia or are an expression of other psychopathology such as depression. Such experiences represent an important public health concern however as they are associated with distress and functional impairment (Zammit et al., 2013), and understanding more about their aetiology is of importance irrespective of the extent to which this contributes to understanding the aetiology of disorder such as schizophrenia.

## 7 Strengths and limitations

The strengths of this study are the large sample size and its longitudinal nature. Antenatal life event measures and confounders were measured during pregnancy, not retrospectively. Semi-structured interviews are the gold standard measure for psychotic symptoms. Valid and reliable measures of depression (EPDS), anxiety (Crown–Crisp score) and psychotic experiences were used (Cox et al., 1987, Birtchnell et al., 1988).

Attrition is a potential source of bias in longitudinal studies. If children of mothers who were most stressed during pregnancy and children with psychotic experiences have higher attrition, this is likely to lead to an underestimate of effect. In previous studies of psychotic symptoms and other psychopathology in this sample there has been very little evidence of bias resulting from attrition (Wolke et al., 2009).

It is possible that the life event questionnaire utilised is not a valid measure of life events. Non-differential misclassification may be one explanation for the negative finding in this study. Our maternal life event questionnaire was informed by Brown and Harris' life event measure, but has a different construction (Brown et al., 1973a). However, life event questionnaires similar to those we have used have been found to have associations with later mental illness in previous studies (Jordanova et al., 2007, Araya et al., 2009). This suggests that our questionnaire may be valid. Interestingly, our binary life event ratings with no subjective scores or weightings have an equally strong association as our subjective (weighted/unweighted) scores. This suggests that the combined number of stressful life events occurring may be as relevant as the perceived severity of individual events in a study of this size.

Due to the broad time period over which women completed each questionnaire, it is possible that there was overlap between events recorded in the two questionnaires. Conducting separate analyses from the early and late pregnancy life events data addressed this possibility.

Life events are not a straightforward environmental measure. Although we are suggesting that depression and anxiety may be acting as mediators, the measures of SLEs and maternal depression and anxiety are cross-sectional. We cannot examine the association in a longitudinal manner to establish with more confidence direction of association between life event exposure during pregnancy and psychotic experiences at 12 years.

## 8 Implications

The absence of an association between life events in pregnancy and later psychotic experiences in offspring, after taking into account antenatal anxiety and depression, does not rule out a role for stress in the development of psychosis. The neural diathesis stress model (Walker and Diforio, 1997) brings together theories of prenatal complications, psychosocial stress, the HPA axis and hippocampal structure and function. This study provides some evidence to suggest that life events during pregnancy may affect child psychotic experiences through effects on maternal psychopathology, and possibly physiology, during pregnancy. Further research is needed to increase our understanding of the role of antenatal stress in aetiology of psychotic symptoms, and of the mechanisms through which this is mediated.

## Role of funding source

The study was funded by NIHR, UK Medical Research Council and the Wellcome Trust.

NIHR funded the 2 year Academic Foundation Doctor Training Programme UK MRC and Wellcome Trust funded the ALSPAC study.

## Conflict of interest

None.
